# Life-Threatening Conditions in Children with Bocavirus Infection-Case Series and Mini Review of the Literature

**DOI:** 10.3390/v16091347

**Published:** 2024-08-23

**Authors:** Elena Tătăranu, Felicia Galos, Liliana Anchidin-Norocel, Roxana Axinte, Florin Filip, Sorin Axinte, Adrian Tătăranu, Monica Terteliu, Smaranda Diaconescu

**Affiliations:** 1 “Sf. Ioan cel Nou” Emergency Hospital, 720237 Suceava, Romaniaroxaxinte@gmail.com (R.A.); florin.filip@usm.ro (F.F.); axintesorin@gmail.com (S.A.); monica.terteliu@usm.ro (M.T.); 2 Faculty of Medicine and Biological Sciences, Stefan cel Mare University of Suceava, 720229 Suceava, Romania; 3 Marie Curie Emergency Children Hospital, 077120 Bucharest, Romania; 4 Department of Pediatrics, Carol Davila University of Medicine and Pharmacy, 020021 Bucharest, Romania; 5 Faculty of Medicine, “Titu Maiorescu” University of Medicine, 031593 Bucharest, Romania

**Keywords:** children, bocavirus, pneumothorax

## Abstract

In this study, we present four cases of Human Bocavirus (HBoV) infection in children aged between 1 month and 4 years. Among these cases, two siblings were hospitalized with similar symptoms. Among the four pediatric cases of patients with HBoV infection, three were associated with acute respiratory failure and spontaneous pneumothorax, and two of these presented with subcutaneous emphysema. The presented patients were young children, aged between 1 month and 4 years, two of whom were siblings, suggesting a possible intrafamilial transmission of HBoV1 infection. These cases highlight the importance of considering HBoV as a differential diagnosis in pediatric patients with respiratory and gastrointestinal symptoms. Early recognition and appropriate medical care are important in treating HBoV infection in young children.

## 1. Introduction

Human Bocavirus (HBoV) is a parvovirus that was isolated two decades ago and primarily affects the lower respiratory and gastrointestinal tracts of children worldwide [1]. It is a small, icosahedral, linear, non-enveloped, single-stranded DNA virus that measures between 18 and 26 nm [2]. The first variant, HBoV1, was identified by Allander in 2005 in respiratory samples of 17 children with acute respiratory infections from Sweden, where it was suspected to be of viral origin [3,4]. Later, in 2009, three other Bocaviruses (HBoV2, 3, and 4) were successively found in fecal samples [5]. The name Bocavirus is a combination of the words “bovine parvovirus” and “canine parvovirus”, which have similar genetic and amino acid structures [6]. HBoV usually occurs in infants and children between 6 and 24 months of age but is sometimes found in children older than 5 years and in adults [7]. HBoV causes a wide spectrum of respiratory diseases in children, including bronchiolitis, the common cold, asthma exacerbations, acute otitis media, and pneumonia [8]. HBoV patients present with nonspecific symptoms such as cough, wheezing, pneumonia, and fever [9]. In addition, acute gastroenteritis may occur, especially with HBoV2 and HBoV3. Rhinorrhea, asthma attacks, and bronchiolitis have been reported in some young patients associated with progressive HBoV infection. Two rare and life-threatening conditions that may occur in children infected with HBoV are pneumomediastinum and bilateral pneumothorax [10]. Here, we present a case series that include several pediatric patients with severe complications due to HBoV infection.

## 2. Materials and Methods

This study is a four-year retrospective analysis with a descriptive focus, examining a series of four pediatric patients who experienced severe complications due to HBoV infection in “Sf. Ioan cel Nou” County Hospital, Suceava, Romania. Informed consent was given by the children’s parents. This study was approved by the Ethical Committee of “Sf. Ioan cel Nou” County Hospital, Suceava, Romania, nr 8675/29.04.2024.

In the Pediatric Section of “Sf. Ioan cel Nou” County Hospital, Suceava, Romania, in 4 years, there were almost 4800 cases of respiratory infection and 5 cases of pneumothorax, of which 3 were with HBoV and 2 were with necrotizing pneumonia with *Staphylococcus aureus*.

In a pandemic context, upon admission to the hospital, a sample of nasopharyngeal aspirate was obtained from patient 1, 2, and 3, using a standardized procedure of the Viral Respiratory Infections Laboratory within the National Institute for Medical and Military Development Research “Cantacuzino”, Bucharest, Romania, for the detection of the genome of respiratory viruses by real-time RT-PCR-multiplex (Influenza viruses type A and B, Syncytial respiratory virus, Parainfluenza viruses, Coronaviruses, Metapneumovirus, Rhinovirus, Bocavirus, Enterovirus, and Adenovirus). In the case of patient 4, the sample collection for respiratory viruses was conducted after 24 h because she was transferred from Pediatric Surgery, but bacterial culture collection was conducted upon admission to the hospital.

The samples collected immediately upon admission eliminated the risk of false-negative culture results since antibiotics had not yet been administered either at the hospital or at home. Also, the results of C-reactive protein, bacterial cultures, and moderate leukocytosis led to viral infection, and this was confirmed by presence of HBoV with RT-PCR.

### 2.1. Case 1

**Figure 1:**
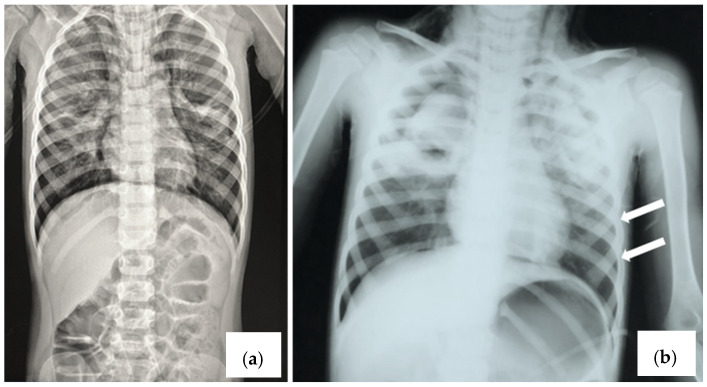
Chest X-ray (anterior–posterior) showing (**a**) diffuse interstitial infiltrate; (**b**) left pneumothorax indicated with arrows.

A 4-year-old boy was admitted in our hospital with a 24 h history of fever, tachypnea, hypoxia, and respiratory failure. He received ibuprofen and antitussive medication at home. The clinical examination revealed perioral cyanosis, dry spastic cough, expiratory dyspnea with tachypnoea (respiratory rate 48/min), respiratory failure, tachycardia (heart rate: 150 beats/min). On auscultation, there were widespread expiratory wheezing and inspiratory crackles. The patient had no significant medical history. The chest X-ray performed at admission showed bilateral interstitial infiltrate and the appearance of interstitial pneumonia (Figure 1a). Laboratory tests revealed the following: leukocytosis at 18.000/mmc, elevated neutrophils PMNs at 63%, and elevated C-reactive protein level CRP at 8 mg/dL, without any other modifications. The initial diagnoses were interstitial pneumonia and respiratory failure.

Broad-spectrum antibiotherapy (ceftriaxone and aminoglycosides) was initiated, together with systemic steroids, antipyretics, short-term bronchodilators, hydroelectrolytic rebalancing, and oxygen therapy. After a few hours, the general condition worsened suddenly; the patient exhibited severe dyspnea with polypnea (respiratory rate (RR): 50–60/min), respiratory failure, inspiratory crackles, and tachycardia (heart rate (HR): 150–165/min). SaO_2_ levels remained low despite oxygen therapy (4–6 L/min), ranging between 85% and 90%, and respiratory acidosis was highlighted in a blood gas analysis (pH 6.81, PaCO_2_ 106 mmHg, PaO_2_ 56 mmHg).

In a short time, bilateral cervical subcutaneous emphysema appeared. The patient was transferred to the intensive care unit where he was intubated and mechanically ventilated, without correcting the blood gas values. The patient exhibited persistent severe respiratory acidosis (pH 6.69). Another chest X-ray was performed, showing massive left pneumothorax and cervical subcutaneous emphysema (Figure 1b).

**Figure 2:**
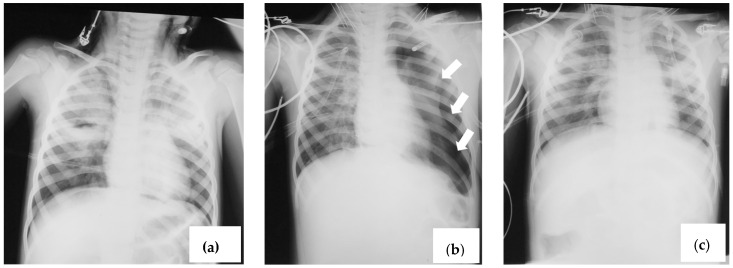
Thoracic X-ray (anterior–posterior) showing (**a**) diffuse interstitial infiltrate, consolidations, cavitation (right), and small pneumothorax; (**b**) large left pneumothorax indicated with arrows; (**c**) lung re-expansion following pleurotomy with exsufflation.

Right pleurotomy and exsufflation were performed without improving oxygenation or blood pH, both of which remained low. After about 15 h, the pneumothorax reappeared, and a left pleurotomy with exsufflation was performed but lung re-expansion was not followed by improvement in oxygenation (Figure 2a–c).

Throughout the patient’s hospitalization in the intensive care unit (ICU), the patient received acid–base and hydroelectrolytic rebalancing treatment, the above-mentioned antibiotic therapy, inotropic support, and sedation. After 48 h from admission, multiple organ failure sets in the biological re-evaluation highlighted the following: AST at 842 U/L (reference range < 36 U/L), amylasemia at 1765 U/L (reference range 15 U/L–115 U/L), creatinine at 6.21 mg/dL (reference range < 0.47 mg/dL), blood urea at 159 mg/dL (reference range < 39 mg/dL), total serum protein at 3.7g/dL (reference range 6–8 g/dL), lactate dehydrogenase at 2461 U/L (reference range 120–300 U/L), and troponin T at 559.9 pg/mL (normal values < 14 pg/mL).

The decision of escalating antibiotic therapy was made considering the patient had no significant clinical response to treatment. The respiratory viral panel PCR was positive only for HBoV.

Nasopharyngeal cultures, tracheal aspirate, and blood cultures were obtained, as well as bacterial culture tests, all of which were negative. On the 3rd day after admission, cardiorespiratory arrest occurred with no response to resuscitation maneuvers and a fatal outcome.

### 2.2. Case 2

A 10-month-old female toddler, sister of the previous patient, presented to our pediatric ER after 14 days after her brother with cough, respiratory distress, wheezing, tachypnea RR-64/min, subcostal and intercostal retraction, and hypoxia with a SaO_2_ level of 86–92%, corrected after administration of O_2_ at a free flow rate of 2–4 L/min. She was hospitalized for acute bronchiolitis with supportive therapy like bronchodilators (mostly β2-adrenergic agonists) and oxygen supplementation.

Her hospital stay was uneventful, and she was discharged after 5 days. Nasopharyngeal swabs were collected from the girl and the parents; the result of respiratory viral panel PCR was positive only for HBoV for the patient and the mother and negative for the father.

### 2.3. Case 3

A 30-month-old boy was referred to our service by a local hospital where he was admitted for fever and dry cough followed shortly by respiratory distress and cervical subcutaneous emphysema. He previously received symptomatic treatment prior to the presentation.

On admission, the patient presented pallor with perioral cyanosis, eyelid edema and cervical subcutaneous neck emphysema, anterior epistaxis, respiratory distress, tachypnea at an RR of 60/min, intercostal retraction, wheezing, inspiratory crackles, tachycardia at an HR of 168/min, and hypoxia at a SaO_2_ level of 86–92%.

A plain thoracic X-ray at admission showed bilateral peribronchial and interstitial infiltrates, left cervical subcutaneous emphysema, and small left pneumothorax.

The laboratory tests indicated leukocytosis (WBC = 17,810/mmc), neutrophilia (PMNs—84%), a slightly elevated CRP level at 3.04 mg/dL (reference range < 0.5 mg/dL), mild respiratory acidosis (pH 7.18), increased PaCO_2_ (52 mmHg), and fluid and electrolyte balance within normal limits. The patient was admitted into ICU and received ceftriaxone, systemic steroids, antipyretics, oxygen therapy, salbutamol, light sedation, and intravenous fluids. We noted a rapid favorable evolution, with significant improvement in subcutaneous emphysema, a drop in respiratory rate to 36/min, and hypoxia corrected to a SaO_2_ level of 94%.

The control plain thoracic X-ray showed only bilateral interstitial infiltrate and the absence of pneumothorax. Pharyngeal and nasal swabs were collected, and the result of respiratory viral panel PCR was positive for HBoV but negative for other respiratory viruses. The patient was discharged after 7 days with complete remission of the respiratory symptoms.

### 2.4. Case 4

**Figure 3:**
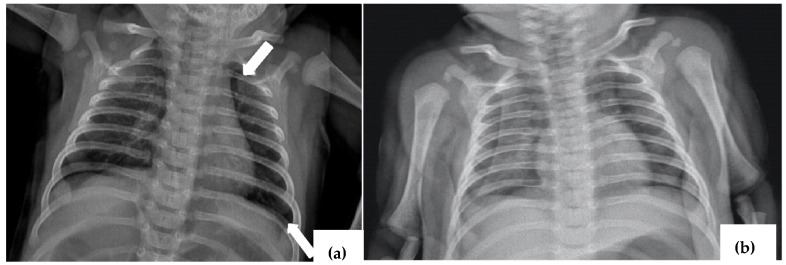
Thoracic X-ray (anterior–posterior) showing with arrows (**a**) small left pneumothorax; (**b**) lung re-expansion.

A one-month old girl was transferred to our service from the Pediatric Surgery department where she was admitted for a radiological image of pneumothorax detected in an outpatient clinic. Her medical history noted cough with an onset of 72 h accentuated in the last 24 h before presentation. The plain thoracic X-ray showed a small left pneumothorax of approximately 2.5 mm (Figure 3), so a drainage procedure was performed.

After chest drainage, the patient presented good general condition, no fever, dry cough, no respiratory distress, SaO_2_-98%, symmetric chest expansion, normal lung auscultation, and a heart rate of 140 beats/min.

Laboratory data revealed WBC = 14,700/mmc with lymphomonocytosis, mild neutropenia PMNs-1200/mmc, mild anemia Hb-11.7 g/dL secondary to inflammation, and CRP within normal limits.

Parenteral steroids and ampicillin/sulbactam together with nebulization with hypersaline solution were started.

**Figure 4:**
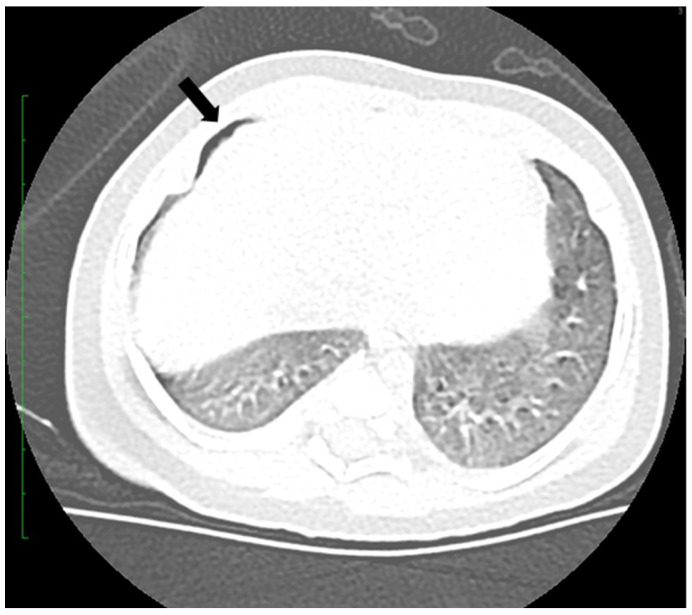
Axial CT scan showing with arrow small right pneumothorax.

After an initial favorable evolution, on the fifth day of hospitalization, the patient developed tachypnea; a plain thoracic X-ray and CT confirmed a right anterior–posterior pneumothorax of a maximum of 2 mm that imposed a new drainage procedure (Figure 4).

The result of the respiratory viral panel PCR was positive for HBoV but negative for other respiratory viruses.

She was discharged fully recovered after 7 days of hospitalization, with normal laboratory data and without radiological detectable pneumothorax.

## 3. Discussion

Pneumothorax is defined by the presence of air between the parietal and visceral pleura [11,12]. This pathology is more common in newborns, particularly those born prematurely, than in other age groups [13]. Pneumothorax, pyopneumothorax, and pneumomediastinum are rare but severe entities in pediatric respiratory pathology [14]. They are most often associated with severe lung infections with multiresistant germs and are an important cause of morbidity in children, being most of the time medical emergencies [15,16]. Spontaneous pneumothorax (SP) that occurs without an apparent cause is divided into primary spontaneous pneumothorax (PSP) and secondary spontaneous pneumothorax (SSP) [17]. PSP occurs in patients without known underlying diseases, while SSP occurs in patients with diseases affecting the lung parenchyma, such as infectious pneumonia, interstitial lung disease, Marfan syndrome, and connective tissue diseases or respiratory diseases such as asthma [4]. Other factors are also considered in the pediatric population, such as cystic fibrosis, necrotizing pneumonia, emphysema of any cause, congenital lobar emphysema, and immunodeficiency [18]. The incidence of SSP is similar to that of PSP and is also more common in males [19]. In children and adolescents, pneumothorax is reported to occur in 4 per 100,000 males and 1.1 per 100,000 females per year [20].

The diagnosis of HBoV infection has so far mainly been based on the detection of viral genomes present in human respiratory samples [21]. The most common methods are quantitative PCR (qPCR) and reverse transcription PCR (RT-PCR) measuring HBoV messenger RNA (mRNA) [22]. Samples from the upper (NP aspirates, NP swabs, or oropharyngeal swabs), middle (tracheal aspirate), and lower respiratory tract (broncho-alveolar lavage) are examined in patients with respiratory tract infection [23]. Almost all routine testing and published studies of HBoV1 infections rely on only PCR testing of respiratory secretions. Many report HBoV involvement in life-threatening respiratory illness in previously healthy pediatric patients [24,25,26,27,28,29,30,31], although the pathogenicity of HBoV as the only etiological agent remains a question [32].

In this report, we presented four pediatric cases, patients with HBoV infection associated with acute bronchiolitis, complicated by pneumothorax, interstitial emphysema, and acute respiratory failure. No other virus or bacterium was detected in the respiratory and blood samples of our cases, which indicates that HBoV is likely to be a true respiratory pathogen that could cause severe and even life-threatening disease. Similar to other viruses, it tends to affect children more during winter months. The most common reason for hospital admission with HBoV infection in children is respiratory distress. According to other research, the most common reason for hospital admission with HBoV infection in children is respiratory distress [7,33].

Clinical features in the four children were mainly represented by lower respiratory tract infection (RTIs), a result also observed in previous research [11,34,35,36,37,38]. The onset was rather nonspecific; two of the patients received symptomatic treatment administrated by their mothers [38]. The majority of patients received oxygen, antibiotics, systemic steroids, and bronchodilators as other authors reported [39,40,41]. All the children in our patient series received at least one antibiotic therapy during hospital admission despite the viral nature of the disease. However, the use of antibiotics was empirical in most of these patients based on the severity of presentation and suspicion of co-existing bacterial infection.

From the four pediatric cases of HBoV infection, three were associated with acute respiratory failure and spontaneous pneumothorax, and two of them presented subcutaneous emphysema, consistent with other researchers’ results [25,42].

All our patients were small-aged children, ranging between 1 month and 4 years old, with two of them being brothers. This suggests a likely intrafamilial transmission of HBoV1 infection, as indicated by the first two cases described and supported by findings from other authors [43].

Most of the pulmonary damage patients that do not recover from ARDS is caused by inflammation and interstitial fibrosis [5,44]. In a small English cohort (29 cases with HBoV mono-infection) reported by Bagasi et al., 38% of patients received oxygen, 31% needed intensive care, and 17% were supported by mechanical ventilation; one patient had a fatal outcome due to multi-organ failure and viral pneumonitis [39].

Another report suggests that HBoV can be the only etiological agent in severe lower RTIs in children; similar to these results, no other virus or bacterium was detected in the respiratory and blood samples of our cases, which indicates that HBoV is likely to be a true respiratory pathogen that could cause severe and even life-threatening disease. There is no clinically approved specific treatment for HBoV infection, and no comparative studies on antiviral drugs have been carried out [45].

The gastrointestinal involvement is currently associated with the variants HBoV 2–4 [46]. The most frequent gastrointestinal symptoms are the loss of appetite and vomiting, followed by diarrhea and nausea [10]. Some controversy surrounds the clinical relevance of these initial findings, because the electron micrographic appearance of parvoviruses can be confused with that of other gastrointestinal pathogens, such as astrovirus [47], and the particles were also found in asymptomatic children [48].

Romanian research regarding HBoV in pediatric patients are scarce, and to the best of our knowledge, this is the first series of pediatric cases reported in our country. For example, the National Institute of Public Health reported only three cases at the national level in 2022 [49]. This study highlights the role of a recently described pathogen (Bocavirus) in the respiratory pathology of young children. The limitations of this research mainly involve the small number of cases. In our opinion, routine testing of children with severe respiratory pathology could bring valuable information on the epidemiology of the infection in our country.

## 4. Conclusions

HBoV bronchiolitis may progress to rare but severe complications, and it should be kept in mind as an etiological agent of respiratory tract infections, especially in children younger than five years old. These aspects suggest the importance of careful monitoring of HBoV infections in young children, especially in the family context, and the need to include HBoV in the differential diagnosis of acute respiratory failure and its complications.

## Data Availability

The data presented in this study are available in this article.
